# diffHic: a Bioconductor package to detect differential genomic interactions in Hi-C data

**DOI:** 10.1186/s12859-015-0683-0

**Published:** 2015-08-19

**Authors:** Aaron T.L. Lun, Gordon K. Smyth

**Affiliations:** 1 The Walter and Eliza Hall Institute of Medical Research, 1G Royal Parade, Parkville, VIC, 3052 Melbourne Australia; 2 Department of Medical Biology, The University of Melbourne, Parkville, VIC, 3010 Melbourne Australia; 3 Department of Mathematics and Statistics, The University of Melbourne, Parkville, VIC, 3010 Melbourne Australia

**Keywords:** Hi-C, Genomic interaction, Differential analysis

## Abstract

**Background:**

Chromatin conformation capture with high-throughput sequencing (Hi-C) is a technique that measures the *in vivo* intensity of interactions between all pairs of loci in the genome. Most conventional analyses of Hi-C data focus on the detection of statistically significant interactions. However, an alternative strategy involves identifying significant changes in the interaction intensity (i.e., differential interactions) between two or more biological conditions. This is more statistically rigorous and may provide more biologically relevant results.

**Results:**

Here, we present the diffHic software package for the detection of differential interactions from Hi-C data. diffHic provides methods for read pair alignment and processing, counting into bin pairs, filtering out low-abundance events and normalization of trended or CNV-driven biases. It uses the statistical framework of the edgeR package to model biological variability and to test for significant differences between conditions. Several options for the visualization of results are also included. The use of diffHic is demonstrated with real Hi-C data sets. Performance against existing methods is also evaluated with simulated data.

**Conclusions:**

On real data, diffHic is able to successfully detect interactions with significant differences in intensity between biological conditions. It also compares favourably to existing software tools on simulated data sets. These results suggest that diffHic is a viable approach for differential analyses of Hi-C data.

**Electronic supplementary material:**

The online version of this article (doi:10.1186/s12859-015-0683-0) contains supplementary material, which is available to authorized users.

## Background

**Fig. 1:**
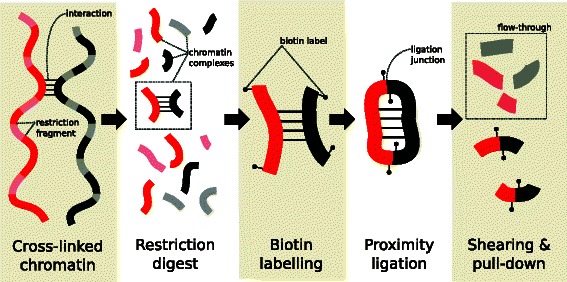
Main steps in the Hi-C protocol prior to sequencing. Chromatin is cross-linked and cleaved by a restriction enzyme. Interacting loci are held together in the same chromatin complex. Restriction fragment ends are filled in with biotin-labelled nucleotides and subjected to proximity ligation and shearing. Biotin-labelled ligation products are purified for paired-end sequencing. For simplicity, the steps after the restriction digest are only shown for one chromatin complex

Chromatin conformation capture with high-throughput sequencing (Hi-C) is a technique that is widely used to study global chromatin organization *in vivo* [1]. Briefly, samples of nuclear DNA are cross-linked and digested with a restriction enzyme to release chromatin complexes into solution (Fig. 1). Each complex may contain multiple restriction fragments, corresponding to an interaction between the associated genomic loci. After some processing, proximity ligation is performed between the ends of the restriction fragments. This favours ligation between restriction fragments in the same complex. The ligated DNA is sheared and purified for high-throughput paired-end sequencing. Each sequencing fragment represents a ligation product, such that each read in the pair originates from a different genomic locus. The intensity of an interaction between a pair of genomic loci can be quantified as the number of read pairs with one read mapped to each locus. The output from the Hi-C procedure spans the genome-by-genome “interaction space” whereby all pairwise interactions between loci can potentially be detected. As such, careful analysis is required to draw meaningful biological conclusions from this type of data.

Most analyses of Hi-C data have focused on identifying “significant” interactions from a single sample [2, 3]. This is challenging because non-specific ligation and apparent interactions can arise from a variety of uninteresting technical causes and rigorous analysis requires a precise quantitative understanding of these artifacts. Identifying biologically interesting interactions from a single sample requires elaborate modeling of the background signal in Hi-C experiments in order to correct for systematic biases due to GC content, mappability and fragment length [3]. Such modeling inevitably involves assumptions and approximations. Furthermore, the interaction space for any single sample will be dominated by conserved features such as topologically associating domains [4]. These may not be of scientific interest when interactions specific to a particular cell type or experiment condition are being sought. An alternative approach is to identify interactions that are significantly different across two or more biological conditions [5–7]. These differential interactions (DIs) are likely to be scientifically relevant because they are directly associated with the biological conditions being studied. A differential analysis is also technically simpler because it involves a like-for-like comparison, where the intensity of the same interaction is compared between samples. The fact that the same genome is present across samples implies that sequence-related genomic biases will be largely constant between conditions and therefore will tend to cancel out during testing. It follows that interaction-specific biases due to GC content, mappability and similar causes will be substantially mitigated.

Although several studies have performed custom analyses to detect differential interactions from Hi-C data [5, 6], there are only a couple of publicly available software packages that can do this type of analysis [7, 8]. HOMER is a command-line software suite that tests for DIs, assuming binomially-distributed counts and using a background model that takes sequence-based and compartmental biases into account [8]. However, HOMER is limited to comparisons between two libraries and does not consider the variability between biological replicates. The binomial assumption means that the tests will only account for sequencing variability. HiBrowse is a user-friendly web-tool implemented in Python [7] that can make comparisons between two experimental conditions. This uses the edgeR package [9] to estimate biological variablity between replicates. However, HiBrowse is implemented as a web-tool and is not practical for high-throughput analyses of large-scale datasets.

Here, we present the diffHic package for rigorous detection of differential interactions. Unlike previous tools, diffHic is able to accommodate complex experimental designs, including paired or blocked designs and those with more than two groups. It does this by accessing the generalized linear model functionality of edgeR [10]. diffHic also estimates biological variability between replicates using quasi-likelihood methods that robustly control the type I error and false discovery rates [11]. diffHic includes functionality to consolidate results at different resolutions while maintaining rigorous error rate control.

In the diffHic pipeline, read pairs are aligned to a reference genome, processed for quality control and counted into bin pairs across the interaction space. Low-abundance bin pairs are filtered out and the remaining bin pairs are normalized with non-linear methods to eliminate complex biases between libraries. Bin pairs are tested for significant differences between conditions using the latest methods in the edgeR package [12]. Careful attention is given to filtering and normalization steps that are sometimes overlooked in existing analysis pipelines. In particular, diffHic provides new normalization methods to removed trended biases that are abundance-dependent. diffHic also implements methods to remove simple scaling biases between libraries and methods to remove genomic biases between interactions and between libraries [2]. diffHic can efficiently handle large datasets.

This article outlines the functionality of the diffHic package. The practical use of the diffHic package is demonstrated with some real Hi-C data sets, for which a number of DIs are successfully detected between conditions. Simulated data is also generated to show that diffHic provides improved sensitivity and error rate control for DI detection, compared to the HOMER software suite.

## Implementation

diffHic is implemented as an R package. The code is written primarily in R, with time-critical functions written in C++ for greater speed. It makes use of a number of core Bioconductor packages [13] such as GenomicRanges, Rsamtools and BSgenome, in addition to edgeR. The pipeline takes a set of name-sorted BAM files [14] as input, and processes them into HDF5 files [15] prior to further analysis. A helper script written in Python is also provided to facilitate read alignment. The analysis can be run interactively through an R session, or it can be automated for batch jobs.

## Results and discussion

### Introduction to the real data sets

The diffHic pipeline can be applied on any Hi-C data set containing biological replicates across multiple conditions, where the aim is to detect DIs between conditions. In the following sections, the use of diffHic will be demonstrated on three Hi-C data sets. Each was obtained from the NCBI Gene Expression Omnibus, with the accession number shown below in parentheses. The first data set is taken from a study on human prostate epithelial cells overexpressing the ERG protein or a GFP control (GSE37752) [5]. The aim of the differential analysis in this study is to detect ERG-induced changes in the chromatin structure. The second data set is taken from a study on human embryonic stem cells (ESCs) and lung myofibroblasts (GSE35156) [4], where the aim is to detect changes between cell types. The final data set is taken from a study on mouse neural stem cells before and after deletion of the *Rad21* gene (GSE49017) [16], which aims to identify changes due to the loss of cohesin activity. Two biological replicates are present for each condition in all studies.

### Read alignment and processing

The first step in a Hi-C data analysis is read alignment to a reference genome. However, this is complicated by the presence of chimeric reads. Recall that a proximity ligation step is performed to construct the Hi-C library. This involves ligating together two interacting DNA fragments from different parts of the genome. A chimeric read is generated when sequencing of the ligation product is performed across the ligation junction. This means that the 5^′^ and 3^′^ segments of the read are derived from distinct genomic loci. Correct alignment of the 5^′^ end is more important than that of the 3^′^ end as the location of the latter is already provided by the mate read. Naïvely performing local alignment of full-length reads will be suboptimal as there is no preference for the proper alignment of the 5^′^ end.

The diffHic package uses a pre-splitting strategy to perform chimeric read alignment. This approach takes advantage of the known “signature” sequence around the ligation junction [6]. The ligation signature is easily derived from the known recognition sequence of the restriction enzyme used for the initial digestion of the chromatin. For example, the *Hin*dIII enzyme has a recognition sequence of AAGCTT with a 4 bp 5^′^ overhang, resulting in a ligation signature of AAGCTAGCTT. Each read sequence containing this signature is split into 5^′^ and 3^′^ segments at the centre of the signature, using the Cutadapt program [17]. Each segment of each read in each pair is then independently aligned to the reference genome using Bowtie2 [18]. This pre-splitting approach outperforms the naïve approach for simulated chimeric reads (Additional file 1: Section 1, Table S1). For both chimeric and non-chimeric reads, pre-splitting also outperforms the “iterative mapping” approach, where each read is truncated to a 5^′^ subsequence and gradually extended from the 3^′^ end until it aligns uniquely [2]. Similar differences are observed when these non-naïve strategies are applied to real Hi-C libraries (Additional file 1: Section 1, Table S2).

Once reads are aligned into BAM files, a number of quality control steps can be applied to remove artifacts. The sizes of the sequencing fragments are estimated by computing the distance of each read to the nearest restriction site in the direction of the read, and summing those distances for both reads in the pair. Fragments with sizes above a default threshold of 600 bp are assumed to result from non-specific cleavage and are discarded [2]. Inward-facing read pairs less than 1 kbp apart are also discarded, to avoid dangling ends from inefficient ligation of (incompletely digested) restriction fragments [19]. Similarly, outward-facing read pairs less than 25 kbp apart are discarded to avoid self-ligation products from those fragments.

For the real data, reads were aligned using the pre-splitting strategy to the appropriate reference genome for each study – mm10 for mouse, and hg19 for human. Read pairs were ignored if the 5^′^ segment of either read was unmapped, had a mapping quality (MAPQ) score below 10 or was marked as a potential PCR duplicate with the MarkDuplicates tool in the Picard suite v1.117 (http://broadinstitute.github.io/picard). Quality control was applied to all remaining read pairs, as described. Any technical replicates were pooled into a single library. Approximately 25–55 % of read pairs were retained in the final libraries.

### Counting into bin pairs

**Fig. 2:**
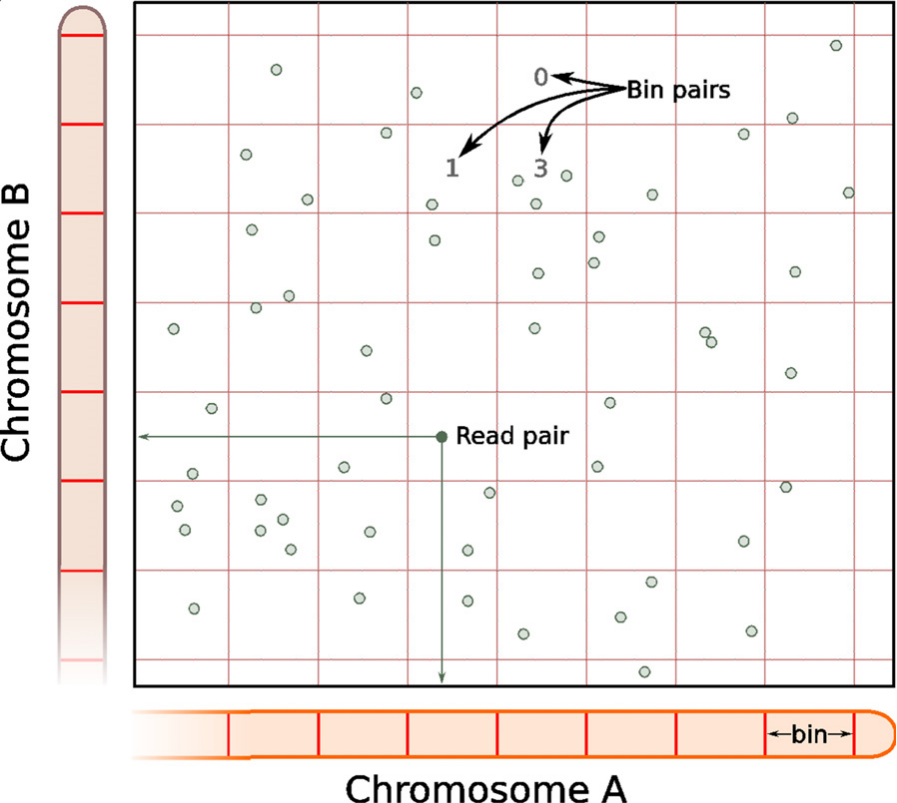
Overview of the counting strategy with bin pairs. The linear genome is partitioned into bins of constant size, such that the interaction space is partitioned into bin pairs (boxes). Read pairs are shown as open circles and are distributed across the interaction space according to the mapping locations of both reads. For example, the marked read pair (closed circle) has one read on each of chromosomes A and B, mapped to the indicated location on each axis. The number of read pairs in each box is used as the count for the corresponding bin pair

After alignment, read pairs need to be summarized into counts for each interaction. A simple binning approach is used here, whereby the genome is partitioned into contiguous and non-overlapping bins of fixed width [1, 5, 6]. Each pair of bins represents an interaction between the corresponding genomic regions. The count for each bin pair is defined as the number of read pairs with one read in each of the corresponding bins (Fig. 2). In this manner, one count is obtained for each bin pair in each library. Note that the boundary of each bin is rounded to the nearest restriction site to reflect the limit of spatial resolution in Hi-C data [2]. The exact location of the interacting locus is largely irrelevant as promixity ligation will always be performed between blunt ends derived from the flanking restriction sites.

The bin size is a critical parameter that determines the desired resolution of the analysis. Larger bins will contain more reads and provide larger counts, increasing precision and power for downstream hypothesis testing [20]. This is often necessary for Hi-C data where read pairs are sparsely distributed across the interaction space. In contrast, smaller bins have lower counts but achieve greater spatial resolution, i.e., adjacent regions in the interaction space can be distinguished. This is important for detecting sharp events such as looping interactions, where the use of larger bins would result in “contamination” by irrelevant counts in the neighbouring space. Traditionally, bin sizes from 100 kbp to 1 Mbp have been used [1, 5, 6, 20] though sizes below 10 kbp are feasible with higher-resolution studies [19, 21]. Analyses with different sizes can be consolidated later for comprehensive detection of DIs.

For the real data sets, pairs of 1 Mbp bins were used for counting. This ensures that the counts are sufficiently large, albeit at the cost of spatial resolution. In addition, bin pairs with one or more bins on chromosome Y were discarded. This avoids spurious detection of DIs between conditions due to sex differences. diffHic is also capable of performing higher-resolution analyses – some results with smaller bin sizes (20–100 kbp) are presented throughout Additional file 1.

### Filtering out low-abundance bin pairs

Filtering is recommended to remove low-abundance bin pairs prior to further analysis. This decreases the severity of the multiple testing correction; avoids loss of accuracy for statistical approximations at low, discrete counts; and reduces computational work. In edgeR’s statistical framework, the filter statistic for each bin pair is the average log-count-per-million (CPM), i.e., the average abundance across all libraries. This is (roughly) independent of the *p*-value under the null hypothesis, i.e., that there is no difference in counts between conditions [22]. Any bin pair with an average abundance below a specified threshold value can be discarded. The aim is to enrich for false nulls without affecting the type I error rate for true nulls [23].

A number of different filtering approaches are implemented in diffHic. The simplest method uses the median abundance of all inter-chromosomal bin pairs as an estimate of the non-specific ligation rate, and only retains bin pairs with abundances above this estimate. This is motivated by the organization of chromosomes into self-contained territories [24], which limits the number of genuine contacts that can occur between chromosomes. Another strategy involves fitting a trend to the abundance of intra-chromosomal bin pairs against genomic distance, i.e., the distance between bins in each bin pair. A bin pair is only retained if its abundance is greater than the fitted value of the trend. This assumes that most interactions are driven by the compaction of the linear genome into the nucleus [25] which is largely uninteresting. Finally, bin pairs corresponding to high-abundance “peaks” in the two-dimensional interaction space can also be identified [21]. This approach regards diffuse interactions as uninteresting and selects for sharp events instead.

The choice of filtering approach for each analysis depends on the interactions of interest. For example, if the researcher is interested in looping interactions, the peak-based approach may be more useful. In this paper, the simple non-specific method was used for filtering in each real data set. This avoids strong assumptions regarding the definition of “interesting”, as non-specific ligation is obviously uninformative and should be removed. Specifically, filtering was performed to only retain bin pairs with average abundances that were five-fold higher than the estimated non-specific ligation rate. This removes the majority of low-abundance bin pairs that are dominated by non-specific ligation, as these are unlikely to be genuine (differential) interactions. Note that the choice of five-fold is arbitrary – other values can be used so long as the majority of low-abundance bin pairs are removed. Obviously, excessively high thresholds are not ideal as power will be lost from removal of genuine DIs.

### Normalization for library-specific biases

**Fig. 3:**
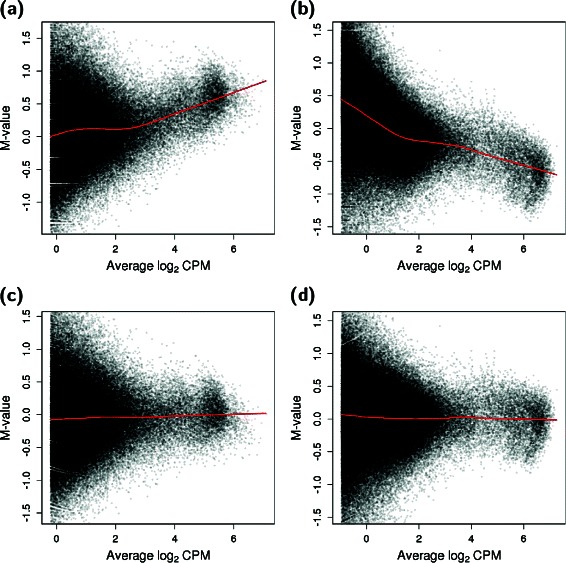
Trended biases with respect to the average abundance for real Hi-C data. Each point represents a 1 Mbp bin pair that is retained after filtering, with a loess trend (red) fitted across all points. The M-value is defined as the library size-adjusted log_2_-fold change between replicates for the ERG-treated cells in the Rickman et al. study [5] (**a**, **c**) or the ESCs in the Dixon et al. study [4] (**b**, **d**). Trends are shown before (**a**, **b**) and after (**c**, **d**) non-linear normalization in diffHic

Library-specific biases can be generated from uncontrolled differences in library preparation. This is particularly problematic for Hi-C data given the complexity of the protocol. Such technical differences may manifest as a trended difference between libraries, where the magnitude of the difference varies as a function of the average abundance (Fig. 3 a, b). An artifactual trend may inflate the variance estimates or fold-changes between libraries, leading to loss of power or spurious differences, respectively. To avoid this, diffHic can perform non-linear normalization using a loess-based method that is adapted for low counts [26]. Its application removes the trends in Fig. 3 c and d, allowing the analysis to proceed safely to statistical modelling. Simple scaling methods [27] are also available, in case the trend represents some interesting biological effect that should not be removed.

**Fig. 4:**
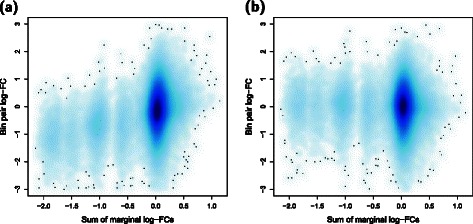
Effect of normalization for CNV-based biases. Each log_2_-FC is defined as that between one ERG library over one GFP library in the Rickman et al. data set [5], adjusted for library size. To simplify visualization, the two marginal log-FCs for each bin pair are summed together. The depth of colour in the plot is proportional to the density of bin pairs, using only those retained after filtering. Results are shown before (**a**) and after (**b**) normalization for CNV-based biases

Copy number variations (CNVs) may also be present between the genomes of cells in different groups. This complicates the detection of DIs, as changes in the interaction intensities due to changes in the copy number of the participating loci are unlikely to be interesting. To avoid detecting these changes, diffHic can eliminate CNV-driven differences in abundance between libraries. This is done by computing the marginal count for each bin (i.e., the number of reads mapped to that bin when Hi-C libraries are treated as single-end) as a proxy for the genomic coverage. The marginal log-fold change (log-FC) is computed between two libraries for each bin, representing the CNV for that bin. Note that this refers to the relative change in copy number between conditions for a given genomic region, not any CNV between regions. For example, chromosomes 10 and 13 are lost upon ERG overexpression, resulting in negative marginal log-FCs (Additional file 1: Figure S1). Each bin pair is associated with two marginal log-FCs as well as its own log-FC between libraries. Multi-dimensional smoothing is applied to all bin pairs [28], whereby a high-dimensional surface is fitted to the bin pair log-FC against the covariates, i.e., the two marginal log-FCs. If the fitted value of the surface changes with the marginal log-FCs across bin pairs, there is likely to be a CNV effect on the interaction intensities. For example, a systematic decrease in the bin pair log-FC with respect to decreasing marginal log-FCs indicates that some CNV-based bias is present in the Rickman et al. data (Fig. 4 a). Normalization of this bias can then be performed based on the fitted value for each bin pair. This results in the removal of the systematic decrease in the normalized bin pair log-FCs (Fig. 4 b). More details on this normalization procedure can be found in Section 2 of Additional file 1.

The iterative correction strategy of Imakaev et al. [2] is also implemented in diffHic. This method factorizes out genomic biases from the interaction intensities, yielding “true” contact probabilities that can be compared between interactions. This method facilitates comparisons between different interactions and can also be used to remove condition-specific genomic biases if these are considered to be important for a particular dataset.

It should be stressed that these normalization strategies do not alter the counts directly. Rather, they compute offsets that are used in fitting generalized linear models (GLMs). For all downstream steps, the offsets computed by the loess-based method (to remove trended biases) were used for the Sofueva et al. data set, while those computed by multi-dimensional smoothing (to remove CNV biases) were used for the Rickman et al. and Dixon et al. data sets. This corrects for the presence of CNVs in the immortalized cell lines that were used in the latter analyses. In all cases, normalization was only applied to bin pairs that remained after filtering.

### Modelling complex experimental designs

Counts for each bin pair are modelled using the GLM methods implemented in the edgeR package [10]. Write *y*_*bi*_ for the count obtained for bin pair *b* in sample *i*. Taking into account the sequencing depth and treatment conditions applied to sample *i*, the expected value of the count can be represented by a log-linear predictor

$$E(y_{bi}) = \mu_{bi} = \sum\limits_{j=1}^{p} x_{ij}\beta_{bj} + o_{bi} $$

where the *x*_*ij*_ are elements of the design matrix specifying which experimental conditions are applied to each sample, and the *β*_*j*_ are unknown coefficients or log-fold changes representing the magnitude of the treatment effect(s). Users specify this log-linear predictor by defining the design matrix in diffHic. For the simplest case involving two experimental groups, the coefficients *β*_*b*1_ and *β*_*b*2_ can be used to represent the log-interaction intensities of the bin-pair in the two conditions. Alternatively, the model can be reparametrized so that *β*_*b*2_ directly estimates the log-fold change in intensity between the two conditions.

The values *o*_*bi*_ are offsets that incorporate the sequencing depth and other normalization factors. The offset *o*_*bi*_ is equal to the logarithm of the total number of unfiltered read pairs for sample *i*, modulated by any normalization factors computed by the methods described in the previous section. The offsets are computed automatically by the diffHic normalization functions and are usually invisible to users. They provide a flexible mathematical means by which bin-specific, condition-specific and sample-specific adjustments can be incorporated into the analysis.

### Modelling technical and biological variability

The variability of the bin-pair count between replicate samples is modeled using the latest quasi-likelihood (QL) methods implemented in the edgeR package [12]. The counts are assumed to follow quasi-negative-binomial distributions, i.e., they are negative binomial (NB) distributed with an additional technical overdispersion parameter. The variance of a count across biological replicates can be written as

$$\text{var}(y_{bi}) = {\sigma_{b}^{2}}\left(\mu_{bi} + \phi_{b}\mu_{bi}^{2}\right) $$

where ${\sigma ^{2}_{b}}$ is the QL dispersion parameter and *ϕ*_*b*_ is the NB dispersion for that bin pair [11]. The value of *ϕ*_*b*_ is estimated by fitting an abundance-dependent trend to the NB dispersions across all bin pairs [10, 29]. Having estimated *ϕ*_*b*_, the value of ${\sigma ^{2}_{b}}$ for each bin pair is estimated by applying a robust empirical Bayes procedure that squeezes individual estimators towards a global trend [11, 12, 30]. The ${\sigma _{b}^{2}}$ vary around unity and represent bin pair-specific variation relative to the average.

**Fig. 5:**
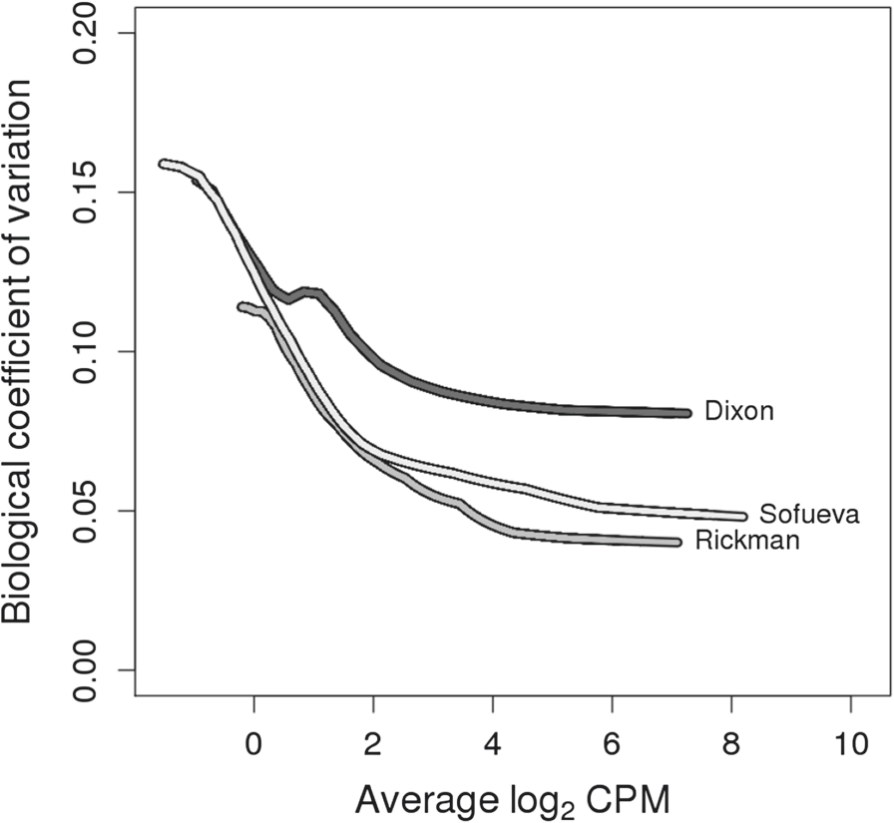
Trended dispersions with respect to the average abundance for real data. The trended NB dispersion was computed using edgeR for each 1 Mbp bin pair in each study, after filtering and non-linear normalization. This was done for the Rickman et al. [5], Dixon et al. [4] and Sofueva et al. [16] data sets. The biological coefficient of variation is defined as the square-root of the NB dispersion, and is shown here for improved resolution of low dispersions

In this model, the NB dispersions *ϕ*_*b*_ represent the level of biological variability between replicates. Specifically, the square root of *ϕ*_*b*_ is the biological coefficient of variation (BCV), i.e., the coefficient of variation with which the count for each bin pair varies between the replicate samples, averaged over bin pairs with similar abundances. It represents the coefficient of variation that would be observed in the counts if the sequencing depth was sufficiently large [10]. A value of *ϕ*_*b*_=0 would imply that only Poisson variation is present between replicates. This is typical of technical replicates formed from repeated sequencing of the same library [31]. In practice, overdispersion is always present between biological replicates due to the additional variability of the biological system, and this manifests as *ϕ*_*b*_>0. Figure 5 shows the estimated BCVs for the real data sets, which varies from about 5 to 15 % depending on the data set and the size of the counts. The decreasing trends with abundance are consistent with similar trends observed for RNA-seq and ChIP-seq data.

### Testing for significant differences

**Fig. 6:**
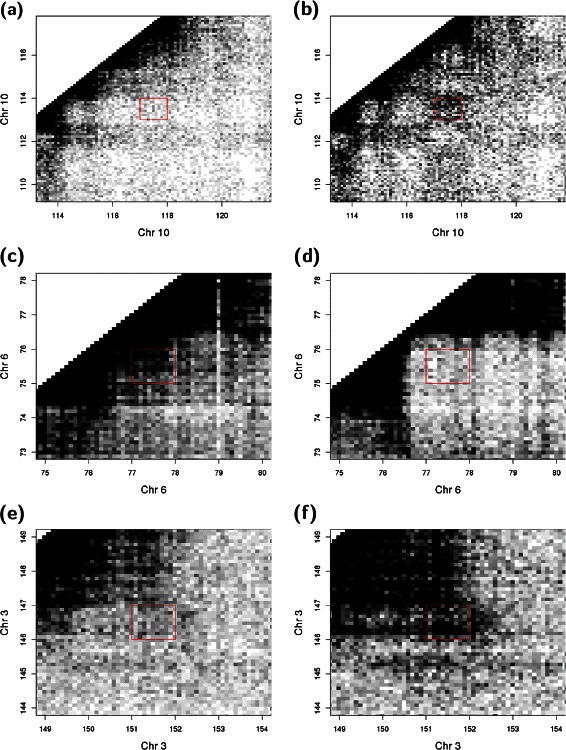
Plaid plots of putative DIs detected in real data. Each “pixel” represents a box in the interaction space with sides of 100 kbp, where the colour of the pixel is proportional to the number of read pairs counted into that box. Putative DIs were defined as 1 Mbp bin pairs detected at a FDR of 5 %. The red rectangle marks the interaction space corresponding to the detected bin pair, in the Rickman et al. data set [5] between ERG- (**a**) and GFP-overexpressing prostate cells (**b**); in the Dixon et al. data set [4] between ESCs (**c**) and lung cells (**d**); and in the Sofueva et al. data set [16] between wild-type (**e**) and *Rad21*-knockout cells (**f**). All coordinates are shown in Mbp. Colours are also adjusted to account for differences in library size

The QL F-test in edgeR can be applied to test for significant differences between biological conditions. This yields a *p*-value for each bin pair, representing the evidence for differential interaction intensities. Correction for multiple testing is performed by controlling the false discovery rate (FDR) with the Benjamini-Hochberg method [32]. Bin pairs corresponding to putative DIs are identified as those with corrected *p*-values below some FDR threshold. Several options are provided to visualize the results, including plaid plots and variations thereof, e.g., rotated plots. Some examples of DIs detected from the real data are visualized in Fig. 6. Validation of several DIs is also described in Section 3 of Additional file 1.

Results can also be consolidated for easier interpretation. If multiple analyses were performed with different bin sizes, smaller bin pairs can be nested within larger “parent” bin pairs. The *p*-values of both nested and parent bin pairs can be combined using Simes’ method [33], yielding a single combined *p*-value that represents the overall evidence for a DI within the parent. The genomic coordinates of the parent bin pair can then be reported, along with the combined *p*-value and its FDR-adjusted value. This avoids redundant results from reporting multiple nested bin pairs individually. Similarly, adjacent bin pairs in the interaction space can be clustered together and reported as a single event to reduce redundancy. This is demonstrated in Additional file 1: Figure S2 for a high-resolution analysis using 20 kbp bin pairs.

### Comparison with existing tools

**Fig. 7:**
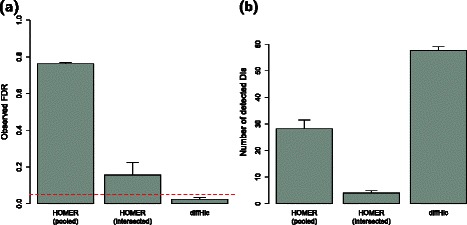
Performance of DI detection methods on simulated Hi-C data. Simulations of multiple groups were conducted with overdispersed counts between biological replicates in each group. Results are shown in terms of (**a**) the observed false discovery rate (FDR) and (**b**) the number of detected true DIs between groups. All values represent the mean of 5 simulation iterations, with standard errors shown as error bars. The nominal FDR threshold is shown as the red dashed line

The presence of overdispersed counts suggests that simple statistical models based on the Poisson or binomial distributions [4–6, 25] will underestimate the actual variance. If these models are used to detect DIs, the significance of any departures from the null hypothesis will be overestimated, i.e., the analysis will be liberal. For example, the HOMER software [8] uses a binomial test to compare counts between samples. Its performance was compared to that of diffHic, using a simulated Hi-C data set with two replicates in each of two groups (see Section 4, Figure S3 in Additional file 1). diffHic controls the error rate for overdispersed counts whereas HOMER does not (Fig. 7 a). One might attempt to mitigate the liberalness of HOMER by only using DIs that were detected in all replicate comparisons. However, this *ad hoc* workaround is not sufficient to restore error rate control (Fig. 7 a). diffHic also detects more DIs than the two other methods (Fig. 7 b), despite its relative conservativeness. These results indicate that the underlying statistical model must properly account for overdispersion to achieve optimal performance.

It should be mentioned that this is not the first time that edgeR has been used to analyze Hi-C data. The HiBrowse pipeline uses edgeR to detect DIs between groups in the presence of biological replicates [7]. However, HiBrowse is limited in that it does not account for trended NB dispersions, complex experimental designs or non-linear normalization schemes. diffHic can naturally accommodate these aspects of the differential analysis, as it uses the latest GLM-based methods in edgeR [10]. diffHic can also account for variable dispersions across bin pairs through the QL framework [11, 12]. Finally, HiBrowse is a web tool that is somewhat inconvenient for high-throughput use, whereas diffHic can be easily run on local systems.

### Intended use and future directions

The diffHic package should be used to detect DIs between two or more biological conditions in a Hi-C experiment. This provides an alternative to conventional analysis strategies that aim to detect “significant” interactions within each sample. The differential analysis may yield more relevant results when the aim of the study is to detect changes in chromatin organization. We anticipate that diffHic – and differential analyses in general – will complement the existing conventional methods, such that the most appropriate analysis strategy can be selected based on the research question. Future development of diffHic will aim to accommodate other types of chromatin conformation data, such as DNase Hi-C [34] and Capture-C [35].

## Conclusions

The diffHic package provides a comprehensive and rigorous pipeline for detecting DIs from Hi-C data. Functions are available for alignment and processing; read counting with bin pairs; filtering of low-abundance bin pairs; normalization to remove trended and CNV-driven biases; statistical analyses to model biological variability and to test for significance; and visualization of detected features. A demonstration with real data provides some examples of the types of DIs that can be detected with this approach. Analyses of simulated data indicate that diffHic provides better performance than the existing HOMER software. These results suggest that diffHic may be a useful alternative to conventional methods for Hi-C data analysis, especially for researchers who want to conduct differential analyses.

## Availability and requirements

The diffHic package is part of the open-source Bioconductor project [13] and can be installed by following the standard Bioconductor installation procedures, as described at http://www.bioconductor.org/packages/release/bioc/html/diffHic.html. diffHic is freely available under version 3 of the GNU General Public License. It is platform independent and can be used on any system that can run R and Bioconductor.

All statistical analyses reported in this article were run on a Dell Precision laptop with an Intel i7 processor and 16 GB of RAM. Analyses were performed using CentOS 6.6, R v3.2.0, Bioconductor v3.1, diffHic v1.0.0 and edgeR v3.10.0. Read alignments were run separately on a Linux server using Bowtie2 v2.2.4. Excluding the Bowtie2 alignnments, all analyses ran in less than an hour using one core.

**Project name:** diffHic**Project home page:** http://www.bioconductor.org/packages/release/bioc/html/diffHic.html **Operating systems:** UNIX, Windows, MacOS Programming language: R version 3.2.0 or higher, C++ Other requirements: diffHic depends on the Bioconductor packages GenomicRanges, Rsamtools, Biostrings, BSgenome, IRanges, S4Vectors, GenomeInfoDb, BiocGenerics, rhdf5, edgeR, limma, csaw, locfit, methods.**License:** GPL-3**Any restrictions to use by non-academics:** none

## Additional file

Additional file 1 **Supplementary materials for the paper.** This PDF file contains a description of the simulation to assess chimeric read alignment strategies (Section 1), comments on the normalization procedure for CNV-based biases (Section 2), some additional validation and high-resolution results and for the real data analyses (Section 3), and a description of the simulation to compare diffHic with HOMER for overdispersed count data (Section 4). Several tables and figures are also included. (PDF 367 kb)
