# Supplementary material for: diffHic: a Bioconductor package to detect differential genomic interactions in Hi-C data
Source: BMC Bioinformatics. 2015 Aug 19;16:258. doi: 10.1186/s12859-015-0683-0 (PMC4539688; doi:10.1186/s12859-015-0683-0)
Supplement: Supplementary file 1 — Supplementary materials for the paper. This PDF file contains a description of the simulation to assess chimeric read alignment strategies (Section 1), comments on the normalization procedure for CNV-based biases (Section 2), some additional validation and high-resolution results and for the real data analyses (Section 3), and a description of the simulation to compare diffHic with HOMER for overdispersed count data (Section 4). Several tables and figures are also included. (PDF 367 kb) [file 12859_2015_683_MOESM1_ESM.pdf]

Supplementary Materials For

diffHic: a Bioconductor package to detect differential genomic interactions in Hi-C data

by

Aaron T. L. Lun[1,2] and Gordon K. Smyth[1,3]

[1]The Walter and Eliza Hall Institute of Medical Research, 1G Royal Parade, Parkville, VIC 3052, Australia

[2]Department of Medical Biology, The University of Melbourne, Parkville, VIC 3010, Australia

[3]Department of Mathematics and Statistics, The University of Melbourne, Parkville, VIC 3010, Australia

21 June 2015

1 Assessing different methods for chimeric read alignment

1.1 Implementation of alignment strategies

Naïve alignment was performed by mapping all full-length reads to the reference genome using Bowtie2 v2.2.4 [20] in local mode at its highest sensitivity. Note that the two reads in each pair were aligned separately in single-end mode. This avoids any inappropriate preference for concordant alignment, i.e., mapping of a read to a genomic position adjacent to its mate.

Iterative alignment was performed as described by Imakaev *et al.* [2], with some practical modifications. Reads were truncated to 25 bp on the $5'$ end. Single-end alignment was performed with Bowtie2 in end-to-end mode at its highest sensitivity. For a valid comparison with the other methods, the custom score function in the original implementation of this approach was not used here. Uniquely mapped reads were defined by the absence of the "XS" tag in addition to a non-zero MAPQ score. Non-uniquely mapped reads were extended by 5 bp on the $3'$ end and realigned. This was repeated for each read until it was uniquely mapped or no further extension could be performed. Only uniquely mapped segments were reported.

The pre-splitting approach identifies the chimeric segments based on the read sequence [6]. The ligation signature for *Hin*dIII (i.e., AAGCTAGCTT) was identified in each read using the Cutadapt program v0.9.5 [19] with the default error rate of 0.1. Reads containing the signature were split into $5'$ and $3'$ segments at the center of the signature. All segments were aligned using Bowtie2 in end-to-end mode at its highest sensitivity. Any unsplit reads were aligned in local mode at the highest sensitivity. Single-end mode was used for all alignment steps.

Note that all of these methods are based on Bowtie2. This provides consistency and ensures that comparisons between the different chimeric alignment strategies are valid.

1.2 Simulations to test alignment methods

Simulated data was generated for a Hi-C experiment in which the *Hin*dIII restriction enzyme was used to digest the hg19 build of the human genome *in silico*. To simulate a chimeric read, two restriction fragments were randomly selected and concatenated together in a random orientation. A random 100 bp interval was chosen that spanned at least 10 bp on either side of the ligation junction in the concatenated sequence. For a non-chimeric read, a random 100 bp interval was chosen within the boundaries of a random restriction fragment. To simulate incomplete digestion, a random 100 bp interval was chosen on the linear genome that contained the flanking 10 bp on both sides of a restriction site. For non-specific digestion, the simulation was performed as described for the chimeric reads. However, both of the selected restriction fragments were randomly truncated prior to concatenation. In all scenarios, the read sequence was defined as the sequence across the chosen interval on a randomly selected strand.

Sequencing errors were then added to the chosen sequences. The quality score for each base was sampled from a scaled $\text{Beta}(5, 2)$ distribution to obtain values in $(0, 40)$. The probability of a sequencing error at a base with a quality score of Q was defined as $10^{-Q/10}$. Errors were introduced by replacing the original base with a randomly chosen alternative in $\{A, C, G, T\}$. Sequences and quality scores were generated in this manner for 100000 read pairs. For simplicity, each mate read was set as the reverse complement of the first read in each pair.

To assess performance, each alignment method was applied to the simulated reads. Reads with MAPQ scores below 10 were ignored. An aligned read or read segment was considered to be correctly mapped if it overlapped the restriction fragment from which the sequence of that read/segment was derived. Otherwise, it was treated as incorrectly mapped. For undigested reads, the relevant restriction fragment was defined as that in which the $5'$ end of the read was located. For chimeras, mapping was assessed using the restriction fragment from which the $5'$ chimeric segment was derived. For unsplit chimeras, failure to map to the fragment of the $5'$ segment was not treated as an error if the read mapped correctly to the fragment of the $3'$ segment. This outcome is neither correct or incorrect in terms of $5'$ alignment.

1.3 Pre-splitting provides consistently high performance

Low error rates are observed for all methods in most scenarios (Table S1). This is consistent with MAPQ filtering such that only confident alignments are retained. It also means that recall can be directly compared between methods. In particular, this simulation focuses on correct alignment of the informative 5′ end of each chimeric read. The lowest recall for these reads is observed for the naïve method. This is expected as alignment of the 5′ end must compete with that of the 3′ end. Thus, any alignment of the former will be ignored in favour of a stronger alignment for the latter. For pre-splitting, the two ends are split into separate sequences to avoid competition during alignment. For iterative mapping, alignment focuses exclusively on the 5′ end. These strategies improve alignment of the 5′ end relative to the naïve approach.

Iterative mapping has lower recall than pre-splitting for chimeric, non-chimeric and undigested reads. This may be due to its stringent definition of uniqueness. A read is only considered as uniquely mapped (and reported) if there are no alternative alignments in the genome [2]. This is necessary to prevent incorrect alignment and premature termination at earlier iterations, but will reduce alignment power. In contrast, pre-splitting avoids multiple mapping by filtering on MAPQ scores. This is more graduated as it accounts for the strength of the alternative alignments. The primary alignment will still be retained if the alternatives are weak, which means that greater recall can be obtained at a similar error rate. Pre-splitting also attempts to identify the exact position of the ligation junction in chimeric reads, whereas the iterative method does not. Thus, the pre-split segments will have more appropriate sequences for alignment.

For non-specific chimeras, all methods except the iterative approach perform poorly. These chimeras are formed independently of restriction sites, so splitting on restriction sites/sequences will not provide any advantage over the naïve approach. The iterative method performs well as it functions independently of restriction site information. This may be useful for DNase Hi-C [35] where no restriction enzymes are used. That said, in standard Hi-C, most non-specific chimeras will not be used for downstream counting, even if they are successfully mapped. This is due to the removal of read pairs with large fragment sizes during quality control.

1.4 Similar results are observed for real data

Reads from several real Hi-C libraries were mapped to an appropriate reference genome using each of the non-naïve alignment methods. Only alignments with MAPQ scores of at least 10 were considered as mapped. The number of read pairs with both 5′ ends mapped was then counted for each method. For iterative mapping, all aligned reads were treated as mapped 5′ ends. For pre-splitting, aligned unsplit reads or split reads with aligned 5′ segments were treated as mapped 5′ ends. Fewer reads were aligned with the iterative mapping approach compared to the pre-splitting approaches (Table S2), consistent with the simulation results. Chimeric read pairs were also identified as those containing one or more split reads after pre-splitting, and made up 5 to 40% of all read pairs in the dataset. Of these, fewer than half were useful, i.e., both 5′ ends mapped. This is consistent with the difficulty of aligning short read segments.

Note that the pre-splitting method can also align the 3′ segment of a chimeric read. This is useful for real data, where the mapping location of the 3′ end should be adjacent to that of the 5′ end of the mate in the same pair. Any inconsistencies are indicative of mapping errors and can be used as a quality control measure during alignment. In these data sets, informative chimeric pairs were identified as those with both 5′ ends mapped and at least one mapped 3′ end. Inconsistencies in chimeric alignment were identified if the mapping position of the 3′ segment of a read was more than 1 kbp away from the position of the 5′ segment of its mate (if split) or that of the whole mate read (if unsplit). Fewer than 5% of these informative pairs were inconsistent (Table S2), indicating that alignment was generally successful.

1.5 Potential drawbacks with pre-splitting

Pre-splitting is more sensitive to sequencing errors that may compromise the identification of the ligation junction. Some robustness is provided by error-tolerant matching to the ligation signature [19]. Unsplit reads are also aligned locally to provide an opportunity for correct

alignment of the $5'$ end, in cases where the signature is overlooked in a chimeric read. The opposite problem arises when the signature is short, poorly defined and/or occurs frequently in the genome. This may result in spurious identification of the signature and unnecessary read splitting. Thus, the nature of the signature should be considered when choosing a restriction enzyme. Most published datasets use the *Hin*dIII restriction enzyme, which generates a well-defined 10 bp ligation signature with few endogenous matches in the human genome.

The rarity of these endogenous matches can be quantified with a few calculations. Consider that there 71196 matches to the *Hin*dIII ligation signature (allowing for 1 mismatching base) in the hg19 build of the human genome. Of these, 27209 lie within 600 bp of a *Hin*dIII restriction site. These matches are most likely to be captured in a Hi-C library after proximity ligation and shearing, assuming that most sequencing fragments are shorter than 600 bp. More distal matches are unlikely to be sequenced, as a short sequencing fragment will not be able to span both the matching location and the ligation junction derived from a restriction site. Now, consider that there are 846132 restriction sites in the entire genome. This means that the endogenous matches will affect less than 5% of the reads (assuming uniform coverage across restriction sites). Thus, the potential for confusion between endogenous and artificial signatures will be limited.

2 Normalization for CNV-based biases

2.1 Detailed description of the procedure

For each bin pair, the first and second covariate was defined as the larger and smaller marginal log-FC, respectively. This avoids manufacturing any arbitrary distinction between bin pairs with different permutations of marginal log-FCs. Of course, normalization could be simplified by summing the pairs of marginal log-FCs into a single statistic. This is not done here to maintain some flexiblity when modelling the biases. For example, the effect of doubling the copy number in both interacting loci in a bin pair can be distinguished from the effect of a quadrupling in one locus only, as the two events would be separated in the covariate space. Also, the marginal log-FC does not need to be an accurate estimate of underlying CNV in that bin. Relative differences are sufficient to correctly separate covariates during loess fitting.

To normalize for CNV-driven biases, a multi-dimensional surface was fitted to bin pair log-FCs against the marginal log-FCs and average abundances using the locfit package v1.5-9.1 [29]. Obviously, the marginal log-FCs are needed as covariates to account for any CNV effect. The average abundance is included as an additional covariate to simultaneously normalize for trended biases. Fitting was done separately for each library, where each log-FC value was computed by comparing that library against an “average” reference library. The reference itself was formed by taking the average count for each bin pair/bin across all libraries. For any given library, the offset for each bin pair was defined as the value of the fitted surface at that bin pair.

2.2 Relating the marginal log-FC to the relative CNV

Karyotyping data is available for both conditions in the RWPE1 study [5], allowing evaluation of the marginal log-FC as an estimate of the relative CNV between conditions. Briefly, over-expression of ERG results in the replacement of one copy of chromosome 13 with a derivative, as well as the (relative) loss of one copy of each of chromosomes 10, 15 and 18. The loss of chromosome 10 upon ERG overexpression is reflected in the negative values for the marginal log-FCs (Figure S1). Similarly, the derivative chromosome has most of the long arm of chromosome 13 replaced with that of chromosome 15, which manifests as negative marginal log-FCs for chromosome 13. No decrease is observed for chromosome 15 itself, possibly because the translocated portion in the derivative compensates for the loss of a copy. In addition, no decrease in the marginal log-FCs is observed for chromosome 18. This may be due to subclone heterogeneity, where a gain of chromosome 18 in GFP-overexpressing cells is only observed for one clone (or specifically, the 10 sampled cells of that clone). Hi-C measurements represent the population average where sporadic gains in one clone would be lost. Indeed, a representative

whole-chromosome painting image in Figure S5 of the original paper does not capture the loss of chromosome 10, which suggests that some heterogeneity is present in this population.

2.3 Further comments on strengths and assumptions

The above procedure is useful as it avoids making assumptions about the factorisability of CNV-driven biases. For example, if there was a doubling in the copy number of two bins, one might expect that the interaction intensity between those bins would quadruple between libraries. However, this reasoning only holds under certain interaction models, e.g., random ligation. If, say, the doubling was due to a change in chromosome number, each chromosome copy might form its own territory in the nucleus [25] such that the count for an intra-chromosomal bin pair would only be doubled. Assuming quadrupling would lead to over-correction during normalization. Here, the use of a fitted surface means that the effect of CNVs on interaction intensity can be empirically estimated. This avoids the need to construct a theoretical model to predict the effect. In the previous example, quadrupling due to random ligation would be detected in the part of the covariate space corresponding to low-abundance bin pairs, while the doubling of intra-chromosomal interactions would be detected separately at higher abundances. Appropriate offsets can then be computed for each bin pair, depending on its covariates.

The implicit assumption of this approach is that the interaction intensity per copy is constant between libraries for most bin pairs at any point in the covariate space. In other words, most of the underlying chromatin structure is assumed to be constant, such that any systematic differences in intensity can be attributed to CNVs and removed without loss of genuine DIs. This may not be true for large-scale genomic rearrangements in, e.g., cancer genomes. CNVs of the same magnitude are also assumed to have the same effect on intensity at any given abundance. This is because only one fitted value is computed for each combination of covariates, such that multiple biases cannot be simultaneously removed. Finally, each marginal log-FC is assumed to be a monotonic function of CNV in the corresponding bin, i.e., an increase in copy number leads to an increase in the marginal log-FC for the affected library against the reference. This assumption is generally reasonable but may not hold when many strong CNVs are present.

3 Additional results for the real data analyses

3.1 Validation of detected DIs

In the RWPE1 data set, several detected DIs were validated with 3C-qPCR (chromatin conformation capture with quantitative PCR) and/or FISH (fluorescence *in situ* hybridisation) in Figure 4 of the original paper [5]. Of particular interest are the interactions between the genes *MOXD1*, *FYN*, *HEY2* and *SERPINB9*. From the 3C-qPCR validation, the *MOXD1-HEY2* and *MOXD1-FYN* interactions decrease and increase in intensity, respectively, upon ERG overexpression. This is also observed in the DI analysis with log-fold changes of -0.82 and 1.54 and p-values of 1.35×10^{-5} and 4.56×10^{-7}, respectively. From the FISH validation, the increase in *HEY2-FYN* contacts is recapitulated in the DI results (log-fold change of 0.73, p-value of 6.59×10^{-3}). However, the DI analysis failed to detect any change in the *MOXD1-SERPINB9* contacts. This is due to the presence of low counts for this interaction (7 and 3 read pairs in the smaller libraries), which limits the available evidence. The original analysis was able to detect this DI as it used Fisher's exact test [5], which does not account for overdispersion. Some loss of power is to be expected when using the relatively conservative NB framework in diffHic.

In the neural stem cell data set, several detected DIs were validated with FISH in Figure S23 of the original paper [18]. FISH results were reported in terms of distance between probe locations, where a decrease in distance is assumed to correspond to an increase in contacts. For comparison with DI results, probe coordinates were transferred from mm9 to mm10 using the UCSC liftOver tool. Some of the interactions were highly local and could not be resolved with 1 Mbp bins, so a differential analysis with 100 kbp bins was used here instead. This also corresponds with the average length of the probes (around 200 kbp). The DI analysis recovered the increase in contacts between probes A and E upon *Rad21* deletion (log-fold change of 3.06,

p-value of 4.82×10^{-10}) as well as the decreases between probes B and D (log-fold change of -0.34, p-value of 2.98×10^{-2}) and C and D (log-fold change of -0.86, p-value of 2.14×10^{-10}). However, the validated decrease in contacts between probes F and G was not observed in the DI results. This is not considered to be a shortcoming of the diffHic analysis. Examination of Figure S23B indicates that the same interaction has a near-zero log-fold change between conditions.

For the human ESCs and lung myofibroblast data set, no direct validation for these DIs was available in the original paper. Thus, no comparisons were performed with DI results.

3.2 Detection of high-resolution interactions

To demonstrate the high-resolution capabilities of diffHic, the DI analysis was repeated for the neural stem cell data set using a bin size of 20 kbp. Normalization and statistical modelling was performed as previously described. Adjacent bin pairs in the interaction space were aggregated into clusters to reduce redundancy in the results. Clustering was performed using a simple single-linkage algorithm, where any adjacent bin pairs were placed into the same cluster. Clusters were restricted to a maximum size of 100 kbp in any dimension, to mitigate chaining effects. The p-value for each cluster was computed from the constituent bin pairs using Simes' method [34], and the FDR was controlled across clusters at 5%. Detected DIs are shown in Figure S2.

4 Comparing diffHic to HOMER

4.1 Simulation design for Hi-C data

Consider a single chromosome in a simulated genome, formed by concatenating n_0 10 kbp blocks. These blocks are partitioned into 160 contiguous, non-overlapping TADs. Each TAD i starts at block s_i and ends at e_i. The width of TAD i is defined as $e_i - s_i + 1$. Partitioning is performed such that widths are distributed uniformly across integer values in the $[10, 30]$ interval.

The interaction space for the simulated genome can be described in terms of pairs of blocks. Consider a lower triangular matrix of order n_0 where each row or column represents a block in the genome. Each entry (x, y) represents an interaction between a pair of blocks x and y, where $x \geq y$ is enforced to avoid redundant references to (x, y) and (y, x). The mean number of read pairs assigned to the block pair (x, y) in a sample from group g is defined as

$$\mu_{(x,y)g} = \begin{cases} 100 & \text{if } x = y \\ k_t(x - y + p)^c & \text{if } x, y \in [s_i, e_i] \text{ for any } i \\ 0 & \text{otherwise} \end{cases}$$
$$+ \quad 5 f_{\mathcal{S}}(x, y)$$
$$+ \begin{cases} k_d(x - y + p)^c & \text{if } (x, y) \in \mathcal{G}_g \\ 0 & \text{otherwise} \end{cases} .$$

In the first condition of this expression, the first case reflects the strong interactions between adjacent loci in the same block. The second case simulates interactions within TADs, given a constant scaling factor $k_t = 80$, prior value $p = 1$ and decay rate $c = -0.8$. This recapitulates the power-law relationship between distance and abundance that is observed in real data [1].

The second condition of the expression represents weak non-specific ligation events that are present throughout the interaction space. The sequence $\mathcal{S}$ was formed by randomly selecting 200000 block pairs from all (x, y) where $x \neq y$. The probability of selecting the pair (x, y) was proportional to $(x - y + p)^c$, to maintain the distance-abundance relationship. The function $f_{\mathcal{S}}(x, y)$ returns the number of occurrences of (x, y) in $\mathcal{S}$. For each occurrence, the intensity of that block pair was increased by a small value to represent the effect of non-specific ligation.

For the third condition in the expression for $\mu_{(x,y)g}$, a set $\mathcal{G}_g$ was constructed for group g by sampling from the set of all block pairs (x, y) where $y + 40 \geq x$. Only block pairs around the diagonal were chosen, given that most real interactions are short-range. In a simulation involving two groups, the sets $\mathcal{G}_1$ and $\mathcal{G}_2$ are of equal size and are mutually exclusive. The

increase in the intensity $\mu_{(x,y)g}$ for any particular $(x, y) \in \mathcal{G}_g$ will generate a DI that is unique to group g. The value of k_d was set to 160, while the size of each $\mathcal{G}_g$ was set to 100. Note that the magnitude of the added intensity from the third condition maintains the power-law relationship.

Finally, the read pair count for each block pair (x, y) in group g was sampled from a NB distribution with a mean of $\mu_{(x,y)g}$ and a constant dispersion of 0.01. Count sampling was repeated to generate two replicate libraries for each of the two groups $g = 1, 2$. In each library, the sampled number of read pairs was explicitly generated for each block pair. Each read was assigned to a random genomic position within its corresponding block on a randomly selected strand. The coordinates of each read pair were then saved into an appropriate format. Some of this data is visualized in Figure S3 to illustrate the various features of the simulation design.

4.2 Differential analyses with HOMER and diffHic

The HOMER software v4.7 [10] was applied to detect DIs between groups. Simulated data in the "HiCSummary" format was processed into tag directories using the makeTagDirectory command. Replicates in each group were pooled together. A background model was constructed for each pooled library at a resolution of 10 kbp (i.e., 1 block). Significant bin pairs were called as DIs in one pooled library against the other, using the analyzeHiC command with no thresholds on the p-value or z-score. This was repeated after swapping the libraries, such that two p-values were initially obtained for each bin pair – if the bin pair was called in only one of the analyses, then the other p-value was set to unity. The DI p-value was defined by doubling the smaller of the initial p-values for each bin pair, equivalent to the output from a two-sided test. The FDR was controlled at 5% by applying the Benjamini-Hochberg correction to all DI p-values.

Alternatively, DIs were detected in the simulated data by intersecting replicate analyses. Libraries were organized into two pairs, where each pair contained one replicate from each group. A DI comparison was performed between the libraries in each pair, using HOMER as described above. Bin pairs were defined as putative DIs if they were detected at a FDR of 5% in both replicate comparisons. This mimics the method used by Seitan *et al.* [6].

For diffHic, a DI analysis was performed by counting read pairs into bins of size 10 kbp. Filtering was performed to remove bin pairs with average abundances below 0. No normalization was performed as no biases were introduced in the simulation. After dispersion estimation, a p-value was computed for each bin pair with edgeR, and the FDR was controlled at 5%.

For each method, the observed FDR was defined as the proportion of detected bin pairs that were not true DIs, i.e., not in $\mathcal{G}_1$ or $\mathcal{G}_2$. Power was quantified as the number of true DIs that were detected by each method. Simulations were repeated, and the average and standard error of the observed FDR and detection power were computed across simulation iterations.

Table S1: Recall and error rate for each alignment method on each class of simulated reads, defined as the proportion of simulated reads that have correctly and incorrectly mapped 5′ ends, respectively. All values are shown as percentages of the total number of reads.

**Method**	Chimeric		Non-chimeric		Undigested		Non-specific	
	Recall	*Error*	*Recall*	*Error*	*Recall*	*Error*	*Recall*	*Error*
Naïve	39.0	0.3	96.4	0.2	96.5	0.1	38.6	0.8
Iterative	71.1	0.1	84.1	0.0	87.5	0.7	67.1	0.1
Pre-split	83.1	0.1	96.3	0.2	96.3	0.1	38.6	0.8

Table S2: Mapping statistics for the non-naïve alignment strategies on real data. The accession number, reference, and total number of read pairs are shown for each library. The number of pairs with both 5′ ends mapped is shown, along with the number of pairs with one or more chimeric read, the number of chimeric pairs that are informative (i.e., at least one 3′ end mapped) and the percentage of those that exhibit inconsistencies between 5′ and 3′ locations.

**Library**	**Ref.**	**Total**	**Method**	**Mapped**	**Chimeras**		
					Total	Info.	Incon.
SRR493818	[5]	28663499	Iterative	16578504	-	-	-
			Pre-split	22840965	5124235	1551177	2.94
SRR400261	[4]	62468562	Iterative	33625039	-	-	-
			Pre-split	44758551	4339934	198640	4.21
SRR941267	[18]	38276623	Iterative	20452625	-	-	-
			Pre-split	25656044	15743000	4427837	2.12

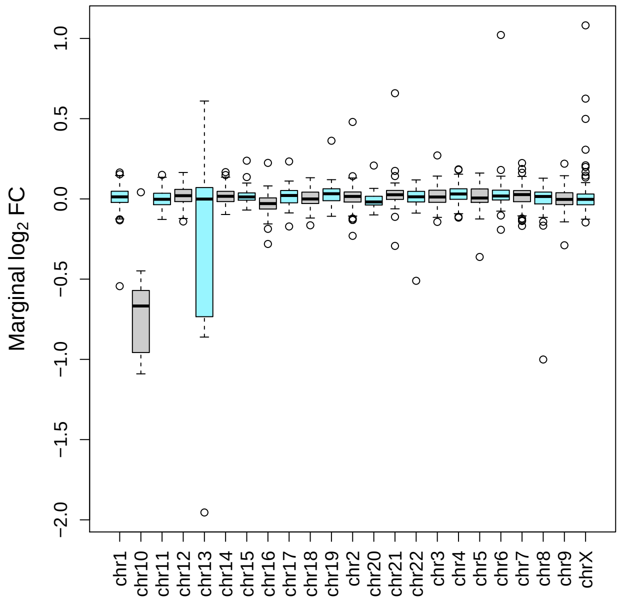

Figure S1: Marginal log-FCs for 1 Mbp bins in each chromosome, for the RWPE1 data set [5]. Each log-FC is computed for the ERG-overexpressing condition over the GFP control, and is adjusted for library size. Outliers are defined as those log-FCs that are more than 1.5 times the inter-quartile range beyond the edge of the boxplot, and are marked with separate points.

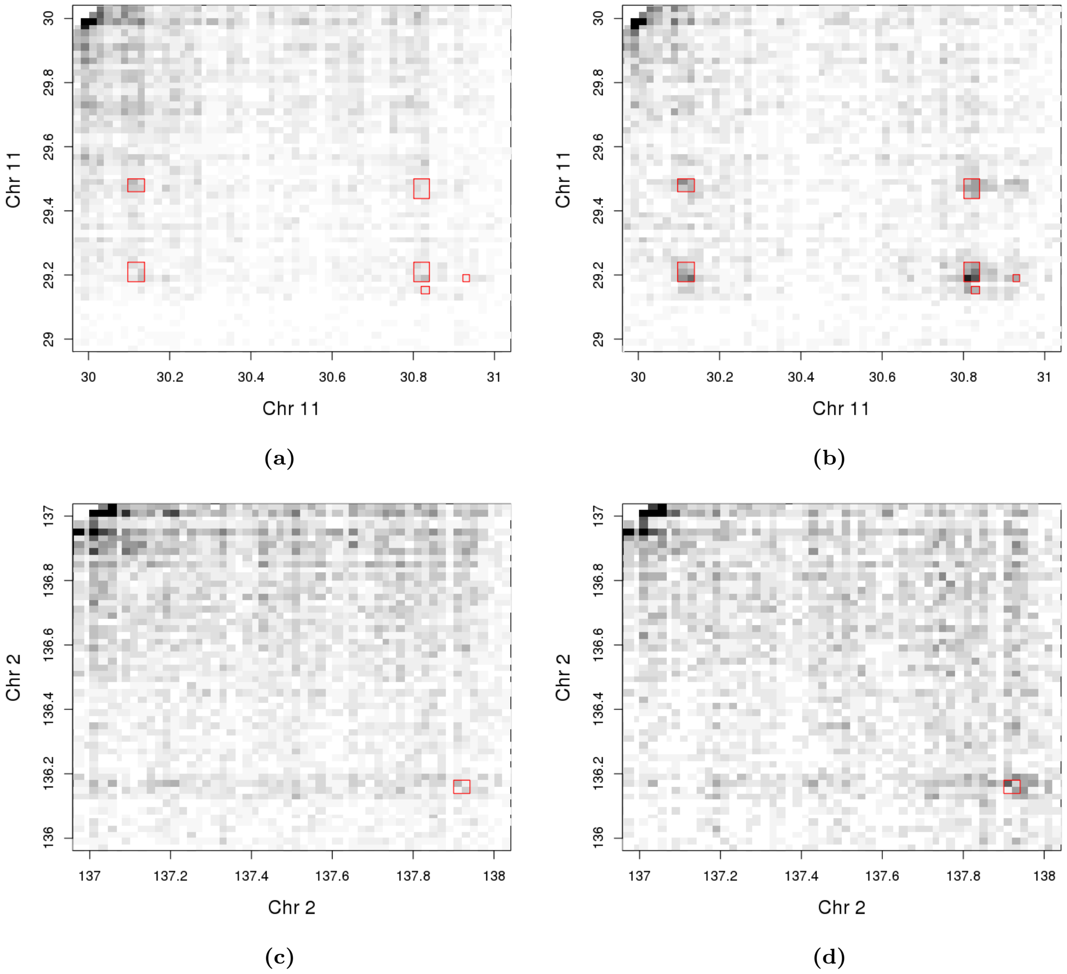

Figure S2: Plaid plots of putative DIs detected in the Sofueva *et al.* data set [18]. Each "pixel" represents a box in the interaction space with sides of 20 kbp, where the colour of the pixel is proportional to the number of read pairs counted into that box. DIs were detected as clusters of 20 kbp bin pairs at a FDR of 5%. Each red rectangle marks the minimum bounding box of a detected cluster in wild-type (a, c) and *Rad21*-knockout samples (b, d). All coordinates are shown in Mbp. Colours are also adjusted to account for differences in library size.

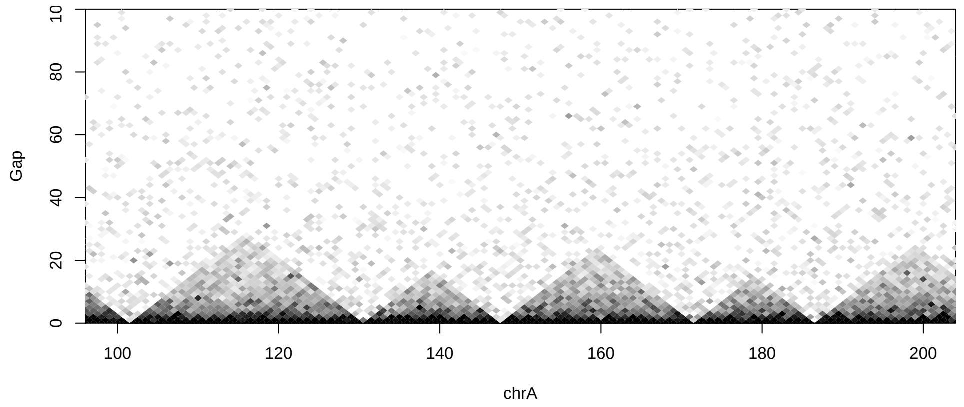

Figure S3: A rotated plaid plot of the simulated Hi-C data for a single library. Each box in the plot represents an interaction between two blocks on the x-axis, locatable by extending diagonals from that box to the x-axis. The intensity of colour corresponds to the number of read pairs connecting those bins in the simulated data. Coordinates are given in terms of blocks, and the value of "Gap" for each box indicates the distance between the interacting blocks.
